# Does Green Tea Ameliorate Obesity in Mice Kept at Thermoneutrality by Modulating Skeletal Muscle Metabolism?

**DOI:** 10.1002/cbf.70094

**Published:** 2025-06-16

**Authors:** Celso Pereira Batista Sousa‐Filho, Marcus Vinicius Aquino Silva, Victória Silva, Kauan Lima, Allanis Valon, Isabela Fiorentino Souza Nascimento, Maria Angélica Spadella, Rosemari Otton

**Affiliations:** ^1^ Interdisciplinary Post‐graduate Program in Health Sciences Cruzeiro do Sul University Sao Paulo Sao Paulo Brazil; ^2^ Human Embryology Laboratory Marilia Medical School Marilia Sao Paulo Brazil

**Keywords:** catechins, green tea, insulin resistance, obesity, skeletal muscle, thermoneutrality

## Abstract

The effects of green tea on metabolic diseases such as obesity and diabetes have been extensively studied. Obesity often leads to insulin resistance, particularly in peripheral tissues such as skeletal muscle. Green tea has shown promise in mitigating insulin resistance in several diet‐induced obesity models. However, its ability to improve insulin sensitivity by modulating skeletal muscle metabolism in the absence of metabolic stress, such as constant cold exposure, remains unclear. Therefore, the aim of this study was to evaluate the effect of green tea on skeletal muscle metabolism in high‐fat diet (HFD)‐induced obese mice maintained at thermoneutrality (28°C). Male C57BL/6 mice were fed a control diet or an HFD for 4 weeks. Then, the HFD group mice were treated with green tea extract (500 mg/kg of body weight) while maintained at thermoneutrality (28°C). At the end of the experimental protocol, we performed metabolic analyses. This study suggested that green tea treatment attenuates the negative effects of HFD by improving muscle fiber cross‐sectional area in the gastrocnemius muscle and increasing the expression of genes involved in lipid metabolism. Although no effect was observed on fatty acid oxidation, green tea improved insulin and glucose sensitivity, as evidenced by glucose and insulin tolerance tests. It also increased the expression of genes associated with glucose uptake and lactate dehydrogenase activity in skeletal muscle. These findings suggest that green tea treatment improves insulin sensitivity by influencing skeletal muscle metabolism even in obese mice maintained at thermoneutrality.

## Summary

The study on the effect of green tea on obesity in mice kept at thermoneutrality is significant as it addresses a less‐explored area in metabolism and nutrition research.

- Maintaining mice at thermoneutrality is crucial as it eliminates the confounding effects of thermal stress that can bias metabolic responses.
- By evaluating the influence of green tea on muscle metabolism under these controlled conditions, the study offers important insights into the potential for improving insulin sensitivity and muscle function in obesity.
- The results suggest that green tea can mitigate the negative effects of a high‐fat diet, making it a potential supplement for dietary interventions.
- This study not only adds to the existing scientific knowledge but also highlights the necessity of considering thermoneutral environments in future studies.

## 1 Introduction

The natural benefits of green tea stem from its rich bioactive compounds, which include free amino acids, caffeine, and polyphenols, especially catechins [1, 2]. Green tea catechins are known for their anti‐inflammatory and antioxidant properties, which help prevent and treat muscle disorders related to aging and other diseases. In skeletal muscle, catechins' effects include reducing myocyte apoptosis, mitigating skeletal muscle protein degradation, and improving lipid and glucose metabolism, as reviewed by Li et al. [3]. In several diabetic rodent models, green tea supplementation has been used to alleviate diabetic conditions; for revision, see Park et al. [4]. Furthermore, investigating the effects of green tea treatment in streptozotocin‐induced type 1 diabetic rats and type 2 diabetic KK‐Ay mice, Ueda et al. demonstrated, in both experimental models, lower plasma levels of fructosamine and glycated hemoglobin. These findings were associated with increased glucose uptake in skeletal muscle and glucose transporter 4 (GLUT4) translocation. Therefore, these studies together contribute to strengthening the hypothesis that green tea treatment may act against hyperglycemia in individuals with obesity and diabetes [5].

Green tea provides health benefits, especially in terms of its antiobesity effects [6, 7]. Studies have revealed that induced obesity in rodents often results in insulin resistance, a common metabolic disorder characterized by impaired glucose uptake. However, these adverse effects can be mitigated with green tea treatment [6, 8]. These studies primarily focus on the impact of green tea on adipose tissue and/or the liver, which are key organs for insulin action. They directly explain the effects of green tea in enhancing insulin sensitivity in rodents fed a high‐fat diet (HFD). Recent findings also indicate that green tea supplementation may alleviate HFD‐induced insulin resistance in rodents, possibly through mechanisms involving increased glucose metabolism and reduced inflammation [9].

Our previous studies have explored the metabolic effects of green tea in different models of diet‐induced obesity. In cafeteria diet‐fed rats, green tea extract supplementation improved glucose homeostasis and modulated key metabolic pathways, leading to enhanced insulin sensitivity [10, 11]. These findings were further supported by in vitro studies, where green tea catechins directly influenced cellular signaling, reducing oxidative stress and inflammatory responses in metabolically active tissues [12]. More recently, we demonstrated that green tea supplementation influences adipose tissue remodeling, contributing to systemic metabolic improvements in obese mice [13]. However, the impact of green tea on skeletal muscle metabolism, particularly under thermoneutral conditions, remained unexplored. It is important to note that all previous studies were conducted in mice kept at standard vivarium temperatures (22°C). This factor may complicate the interpretation of the data obtained when the mice are under thermal stress conditions, as previously described [14]. Therefore, our study is the first to evaluate the effects of green tea under thermoneutral conditions (30°C).

To accurately assess the effects of green tea and minimize external bias from thermal stress on basal metabolism, this study ensured that the mice were maintained in a thermoneutral environment. When studying compounds that may impact energy expenditure, thermoneutral temperature should be considered, as it minimizes additional metabolic stress induced by cold exposure [15, 16]. By creating thermoneutral conditions, we could accurately analyze how green tea influences skeletal muscle metabolism, ensuring that any observed effects are solely due to the treatment and not influenced by environmental factors. Our finding suggests that treating obese mice with green tea in thermoneutral conditions can mitigate systemic insulin resistance and protect against the harmful effects of obesity by modulating glucose metabolism in skeletal muscle.

## 2 Materials and Methods

### 2.1 Animals and Green Tea Treatment

Eight‐week‐old male C57Bl/6 mice were obtained from the University of São Paulo (USP, Brazil). All animals received humane care according to the criteria outlined in the “Guide for the Care and Use of Laboratory Animals.” Also, all mice were housed at thermoneutrality (28 ± 2°C) and 12‐h light/dark cycle, fed with Nuvilab CR1 Radiated diet (standard diet) for a 1‐week acclimation period, and were subsequently divided randomly into the groups: (I) Cont – standard diet + gavage with water; (II) HFD + gavage with water; and (III) HFD + GT (HFD + gavage with green tea). The HFD consisted of 15.18% protein, 27.3% carbohydrates, and 57.20% lipids, with the remaining portion comprising fiber, vitamins, and minerals to total 100%. In contrast, the standard diet contained 22% protein, 43.50% carbohydrates, and 4% lipids, also supplemented with fiber, vitamins, and minerals to reach 100%. The HFD had a caloric density of 531 kcal/100 g, whereas the standard diet had 288 kcal/100 g. Mice received food (standard or HFD) and water ad libitum for 16 weeks. The mice were administered green tea extract from Monday to Friday, starting after 4 weeks on an HFD. A daily dose of 500 mg per kg of body weight was given via intragastric gavage (100 µL), with the extract dissolved in filtered water. The supplementation period lasted for 12 weeks, and the extract was provided each afternoon (between 6:00 and 7:00 p.m.). Green tea extract's total polyphenols content and HPLC analysis of flavonoids and caffeine were as described in our previous study [17]. Briefly, the concentration of polyphenols and catechins in the extract was 52% and 28%, respectively, as obtained by the HPLC analysis and the caffeine content in the extract was 3.4% by dry weight. All procedures in this study followed the ethical principles of animal experimentation as indicated by the Ethics Committee for Animal Experimentation 001_2019 (CEUA) at Cruzeiro do Sul University. At the end of the experimental period, mice were euthanized by exsanguination followed by decapitation between 9:00 and 12:00 a.m. under food state. Tissues were collected, weighed, and stored at −80°C. A total of five mice per group were used in the preclinical trial. The same experimental design was used in a previously published study, where we evaluated the effect of green tea treatment on the profile of immune cells in adipose tissue. Additionally, the data for obesity characterization, including insulin responsiveness via insulin tolerance test (ITT) and glucose tolerance test (GTT), respectively, fasting glucose levels, HOMA‐IR, weight gain, and adiposity index, were also obtained in that same study [17].

### 2.2 Fasting Glycemia, GTT, and ITT

Fasting glycemia was obtained after 8 h of fasting. The GTT and ITT tests were performed on the same mice on different days within the same week, with the GTT conducted on Monday and the ITT on Thursday. GTT was performed to measure glycemia before and after intraperitoneal glucose administration (1 g glucose/kg body weight in 20% solution of glucose in saline 0.9%), glycemia was evaluated 5, 15, 30, 60, and 90 min after glucose injection. ITT test was performed with 6 h of fasting by glucose measurement after intraperitoneal administration of insulin (Humulin R, Lilly, 0.5 IU/kg body weight), following glycemia evaluation in 5, 15, 30, 60, and 90 min. Blood was collected from the tail of the mice, and glucose levels were determined using the Accu‐Chek system (Roche Diagnostics). The area under the curve was calculated for both the GTT and ITT analyses. These assays were performed in a new cohort of animals, following the same procedures described in the previous study [18].

### 2.3 Skeletal Muscle Histology and Biochemical Parameters

For histological analyses, the skeletal muscle was freshly fixed in 4% phosphate‐buffered paraformaldehyde for 24 h. Fixed samples were dehydrated by sequentially increasing ethanol concentrations, cleared in xylene, and then embedded in paraffin wax. The embedded samples were cut into 5 µm sections and stained with hematoxylin and eosin. For skeletal muscle triglycerides (TGs) and total cholesterol quantification, samples (~30 mg) were placed into 1.5 mL‐tubes containing isopropyl alcohol, lysed by ultrasonication in a VibraCell apparatus (Connecticut, USA), centrifuged for 10 min, 10,000×*g* at 4°C and the supernatant was collected and transferred to a new tube, and analyzed using a TG and total cholesterol assay kits. Results were normalized by mg of tissue. Samples were read on the microplate reader Tecan, Infinite 200.

### 2.4 Morphometry of Gastrocnemius Muscle

For each experimental group, a total of 15 histological fields at 200× magnification was randomly captured for measuring the cross‐sectional area (CSA) of muscle fibers. At least 100 fibers per group were analyzed using the closed polygon tool of the CellSens Olympus software (Olympus, Tokyo, Japan), which allows precise outlining of individual fibers to determine their CSA. The data were expressed in square micrometers (µm²).

### 2.5 Glycogen Content and Enzyme Assays

#### 2.5.1 Glycogen Content

Skeletal muscle glycogen content was measured after extraction with KOH and precipitation with ethanol, followed by the determination of glucose through phenol‐sulfuric hydrolysis [19, 20]. Samples (~50 mg) were placed in plastic tubes containing 1.0 mL of 6 N KOH and were incubated in a boiling water bath for 15 min until complete dissolution. Two hundred and fifty microlitres of the homogenate was mixed with 3 mL of 95% ethanol and 100 μL of 10% K_2_SO_4_. A cloudy white precipitate was formed, and the supernatant was discharged after centrifuging (3000 rpm for 3 min). Afterwards, 200 μL of each sample was analyzed at a wavelength of 490 nm on a Tecan spectrophotometric reader (Salzburg, Austria). A standard curve of oyster glycogen (Sigma Aldrich) was prepared for the final quantification, expressed in μmol/weight tissue.

#### 2.5.2 Citrate Synthase (CS) and Lactate Dehydrogenase (LDH) Enzyme Activity

Skeletal muscles were dissected quickly and cut into small pieces before homogenization in accordance with previous studies [21, 22, 23]. Muscles were homogenized with 0.3 mL of extraction medium. The extraction medium for CS contained 50 mM‐Tris and 1 mM‐EDTA adjusted to pH 7.4 with NaOH. The extraction medium for LDH contained 70 mM‐Tris/HCl and 1 mM‐EDTA at pH 8.2. The enzymes were assayed after the homogenate was sonicated for two 15–30 s periods by using a Polytron PTA 20S generator (model PT 10/35; Brinkmann Instruments, Westbury, New York), followed by centrifugation at 7000 rpm, 4°C, then the upper phase was transferred to a new tube. The homogenate was cooled on ice/water during the sonication.

CS was assayed in a medium containing 100 mM‐Tris/HCl, 0.2 mM‐DTNB (5,5′‐Dithiobis (2‐nitrobenzoic acid – Sigma Aldrich)), 1% Triton X‐100 (Sigma Aldrich), 0.1 mM acetyl coenzyme A (Sigma Aldrich), to which 10 µL of homogenate was added. The assay was initiated by the addition of 0.5 mM oxaloacetic acid (Sigma Aldrich). The final pH was 8.1. The total volume was 0.3 mL. Controls were those in which oxaloacetic acid or the sample was omitted. LDH was assayed in a medium containing 50 mM‐Tris/HCI, 10 mM‐sodium pyruvate (Sigma Aldrich), to which 10–20 µL of homogenate was added. The final pH was 7.3. The total volume was 0.3 mL. The assay was initiated by the addition of 0.17 mM‐NADH. Controls were those in which NADH or the sample was omitted. CS activity was assayed by following the rate of change in absorbance at 412 nm and the LDH activity at 340 nm using a spectrophotometer reader (Tecan, Salzburg, Austria). The results were normalized using total protein content, measured by the method of Bradford [24], using bovine serum albumin (BSA) as a standard.

### 2.6 RNA Extraction and Gene Expression Analysis

Total RNA from gastrocnemius skeletal muscle was obtained using TRIzol Reagent, a monophasic solution of phenol and guanidine isothiocyanate, following the procedure proposed by the manufacturer. Initially, 1 mL of the reagent was added to 30–50 mg of tissue. Tissues were homogenized and 0.2 mL of chloroform was added and stirred vigorously for 15 s. Centrifugation at 12,000×*g* for 15 min and at 2°–8°C favored the separation of the RNA phase. The upper aqueous phase containing the RNA was transferred to a new tube and precipitated by adding 0.5 mL of isopropanol. The material was incubated for 10 min at 15°–30°C and centrifuged at 12,000×*g* for 10 min at 2°–8°C. The sediment was washed by adding 75% ethanol, followed by centrifugation at 7500×*g*, for 5 min, at 2°–8°C. The precipitate was air‐dried and dissolved in sterile‐filtered water (Nuclease‐Free Water, Sigma Aldrich) with EDTA at 0.1 mM and heated in the dry bath for 10 min at 55°C. Isolated RNA was quantified using a NanoDrop ND‐ONE‐W spectrophotometer (Thermo Fisher Scientific, Waltham, MA, US), and its integrity was confirmed using agarose gel electrophoresis. Only RNA with guaranteed integrity and OD260/OD280 ratios between 1.8 and 2.0 and phenol‐free was used in the study. RNA was stored at −80°C prior to analysis by real‐time qPCR.

RNA samples (500 ng) obtained from the tissues were transcribed to cDNA in a thermocycler (Veriti, Life Technologies). First, genomic DNA was inactivated by adding DNase (Sigma Aldrich, 5 U/mg) to the diluted RNA of all samples following the manufacturer's instructions. Then, random primers (5 µM) and deoxynucleotide triphosphate (dNTP, 2.5 mM) were added, and the samples were incubated at 70°C for 10 min. Reverse transcriptase enzyme (M‐MLV Reverse Transcriptase, Sigma Aldrich) was then added, and the samples were incubated at 37°C for 50 min. The extracted RNA, converted to cDNA, was used for gene expression analysis. cDNA samples were diluted 1:10 in nuclease‐free water. For the qPCR reaction, EvaGreen qPCR Mix Plus (Solis Biodyne/Sinapse Biotecnology) was used following the manufacturer's instructions. The cycling conditions used were: 12 min at 95°C, and 40 cycles of 15 s at 95°C, 20 s at 62°C, and 20 s at 72°C. Dissociation protocols were used to evaluate the effectiveness of the primers in amplifying the genes specifically. Primer's sequence of target and constitutive genes are presented in Table S1. The delta *C*_t_ method ($2^{∆∆C_{t}}$) was used to calculate relative changes in mRNA abundance.

### 2.7 Western Blot Analysis

Skeletal muscle total proteins were extracted as in the previous study [18]. Briefly, samples were homogenized in a solubilization buffer at 4°C with a Polytron PTA 20S generator (model PT 10/35; Brinkmann Instruments, Westbury, New York). Insoluble material was removed by centrifugation for 20 min at 12,000×*g*. The protein concentration of the supernatants was determined by the Bradford dye‐binding method [24]. Samples containing 30 µg of protein extracts was separated by 10%–12% SDS‐PAGE gel, transferred to nitrocellulose membranes (120 V for 2 h using tank wet‐transfer), blocked in 5% BSA or nonfat dry milk diluted in TBS‐T buffer for 1 h at room temperature and incubated overnight at 4°C under agitation. The antibodies used are presented in Table S2. Detection was performed by Amersham Imager 680 (GE Healthcare Bio‐Sciences AB, Uppsala, Sweden) using Clarity Western ECL Substrate (Bio‐Rad Laboratories). Signals were quantified using UN‐SCAN‐IT gel version 6.1 Software using Amido black staining or anti‐GAPDH as a reference.

### 2.8 Statistical Analysis

Our results are given as mean ± SEM. The variance and the normality of the data were verified by the Shapiro‐Wilk test. Data analysis was performed in GraphPad Prism 8 for Windows. For comparison between the groups, one‐way ANOVA was used, followed by Tukey post‐test to measure significance between means; *p* ≤ 0.05 was statistically significant.

## 3 Results

### 3.1 The Treatment With Green Tea in HFD‐Fed Mice Kept at Thermoneutrality Resulted in Minor Changes in Gastrocnemius Muscle Morphology

**Figure 1:**
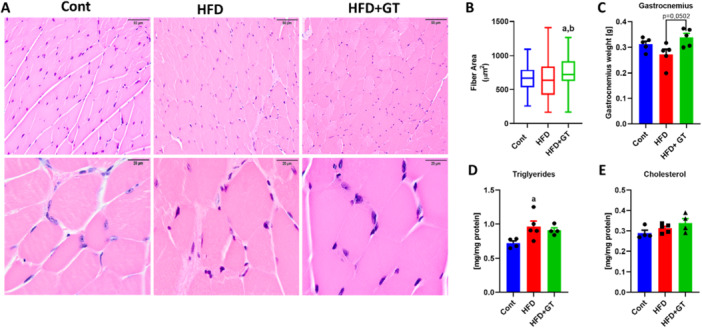
The treatment with green tea in HFD‐fed mice kept at thermoneutrality resulted in minor changes in gastrocnemius muscle morphology. (A) Representative photomicrographs of gastrocnemius muscles from Cont, HFD, and HFD + GT groups, stained with hematoxylin and eosin. (B) Cross‐sectional area (CSA) of the fibers in the gastrocnemius muscles of each experimental group. (C) Gastrocnemius muscle weight (g). (D) Triglyceride (mg/mL) and (E) cholesterol (mg/mg protein) content. Results are presented as mean ± SEM of five animals per group. Statistical analysis was performed using one‐way ANOVA with Tukey's post hoc test, with a significance level set at *p* < 0.05. Superscript letters indicate: a = statistically significant difference compared with the control group, b = compared with the HFD group.

The skeletal muscle used in this study was obtained from obese mice treated with green tea, with data previously published by our group in Tognolli et al. All details regarding obesity characterization, initial and final body weight, weight gain, adiposity index, and other obesity‐related parameters can be found in Figure 1 by Tognoli et al. [17]. In that published study, we observed that HFD‐fed mice treated with green tea showed significant variations in body mass compared to those fed with HFD, suggesting that the treatment effectively prevented body weight increase, even at thermoneutrality. Additionally, treated mice showed reduced feed efficiency and significantly reduced weight gain relative to caloric intake compared to the HFD group. Fasting glycemia in green tea‐treated mice was comparable to that of animals fed a standard diet, while the HFD induced higher fasting glycemia. Glycaemic levels after GTT and ITT were significantly elevated in HFD‐fed mice. Green tea treatment restored levels similar to those of the control mice. Insulin plasma levels and the HOMA‐IR index, indicators of insulin resistance, were similar to the control group in green tea‐treated mice compared to the elevated levels observed in the HFD‐fed mice. These findings indicate that green tea positively affects glycemic control and insulin resistance even at thermoneutrality. With this strong foundation, we proceeded to conduct an initial assessment to determine the impact of green tea treatment on the morphology of skeletal muscle tissue in HFD‐fed mice. The findings indicated a significant increase in the CSA of muscle fibers as a result of the green tea treatment (see Figure 1A,B). We also noted a slight increase in the weight of the gastrocnemius muscle attributed to green tea (see Figure 1C). However, there were no significant changes in cholesterol and TG levels due to the green tea treatment. It is worth mentioning that while we observed an increase in TG levels in the HFD group, there were no changes in cholesterol levels (see Figure 1D,E).

**Figure 2:**
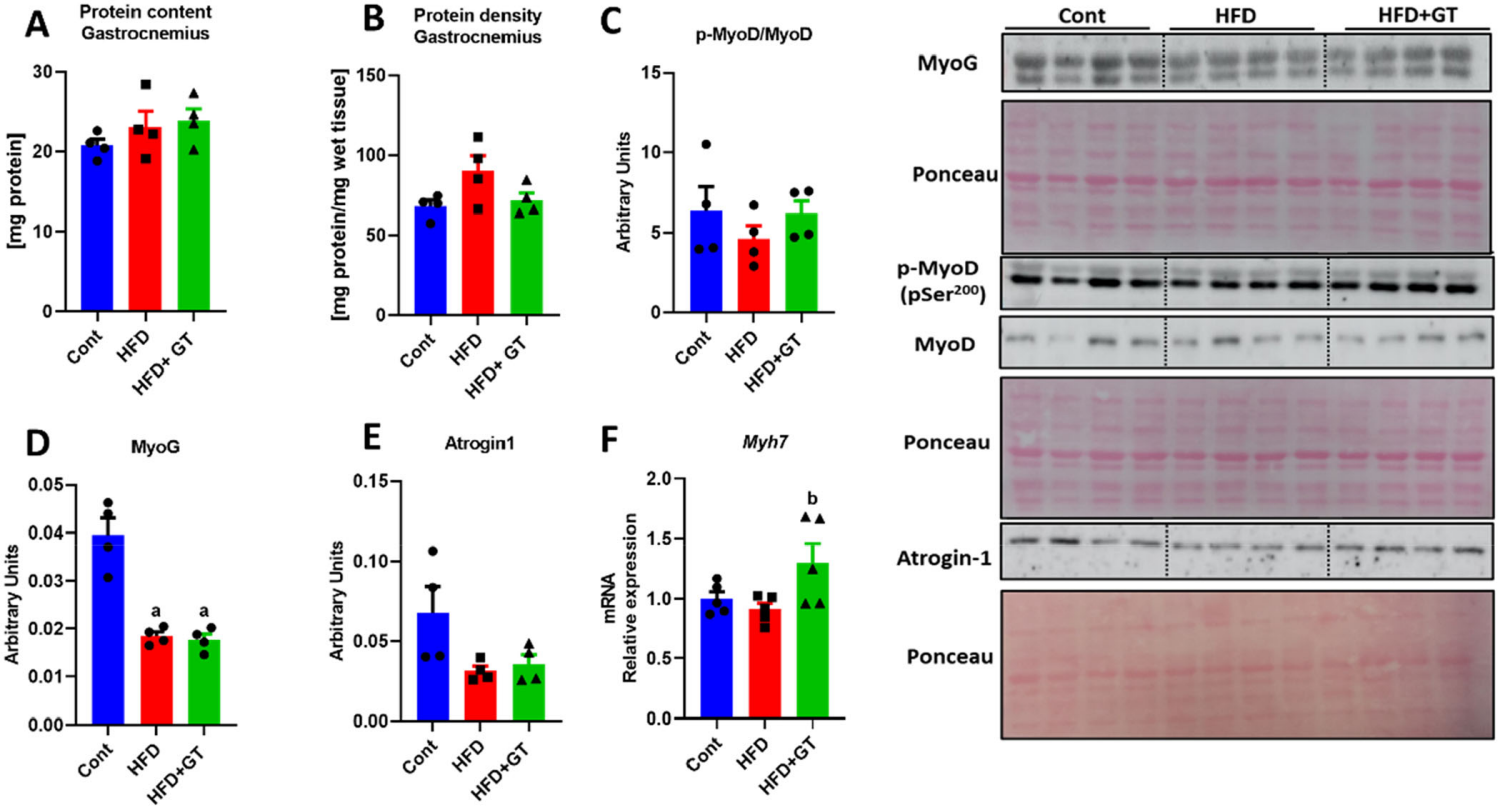
Gastrocnemius protein content and function in HFD‐fed mice treated with green tea kept at thermoneutrality. (A) Total protein content (mg). (B) Protein density (µg protein/mg tissue). (C) p‐MYOD/MYODY ratio analysis performed by Western blot (arbitrary units). (D) Protein content analysis of MYOG and (E) Atrogin‐1 (arbitrary units). (F) *Myh7* gene expression (variation from control). Results are presented as mean ± SEM of four to five animals per group. Statistical analysis was performed using one‐way ANOVA with Tukey's post hoc test, with a significance level set at *p* < 0.05. Superscript letters indicate: a = statistically significant difference compared with the control group, b = compared with the HFD group.

The green tea treatment did not result in significant changes in total protein content or protein density. However, it did positively affect the gene expression of Myh7 (beta‐myosin heavy chain), which encodes a slow‐twitch myosin heavy chain (see Figure 2A,B). Furthermore, the expression of MYOG (myogenin) and MYOD (myoblast determination protein 1), both of which play roles in muscle differentiation, was not significantly influenced by the green tea treatment. Additionally, Atrogin‐1, a marker of muscle atrophy, showed no significant changes either (see Figure 2C–F). Therefore, even though there was an observed increase in muscle fiber CSA, it is evident that this effect was not due to the activation of essential proteins required for muscle function.

### 3.2 Effect of Green Tea on Lipid Metabolism and Muscle Mitochondrial Biogenesis

**Figure 3:**
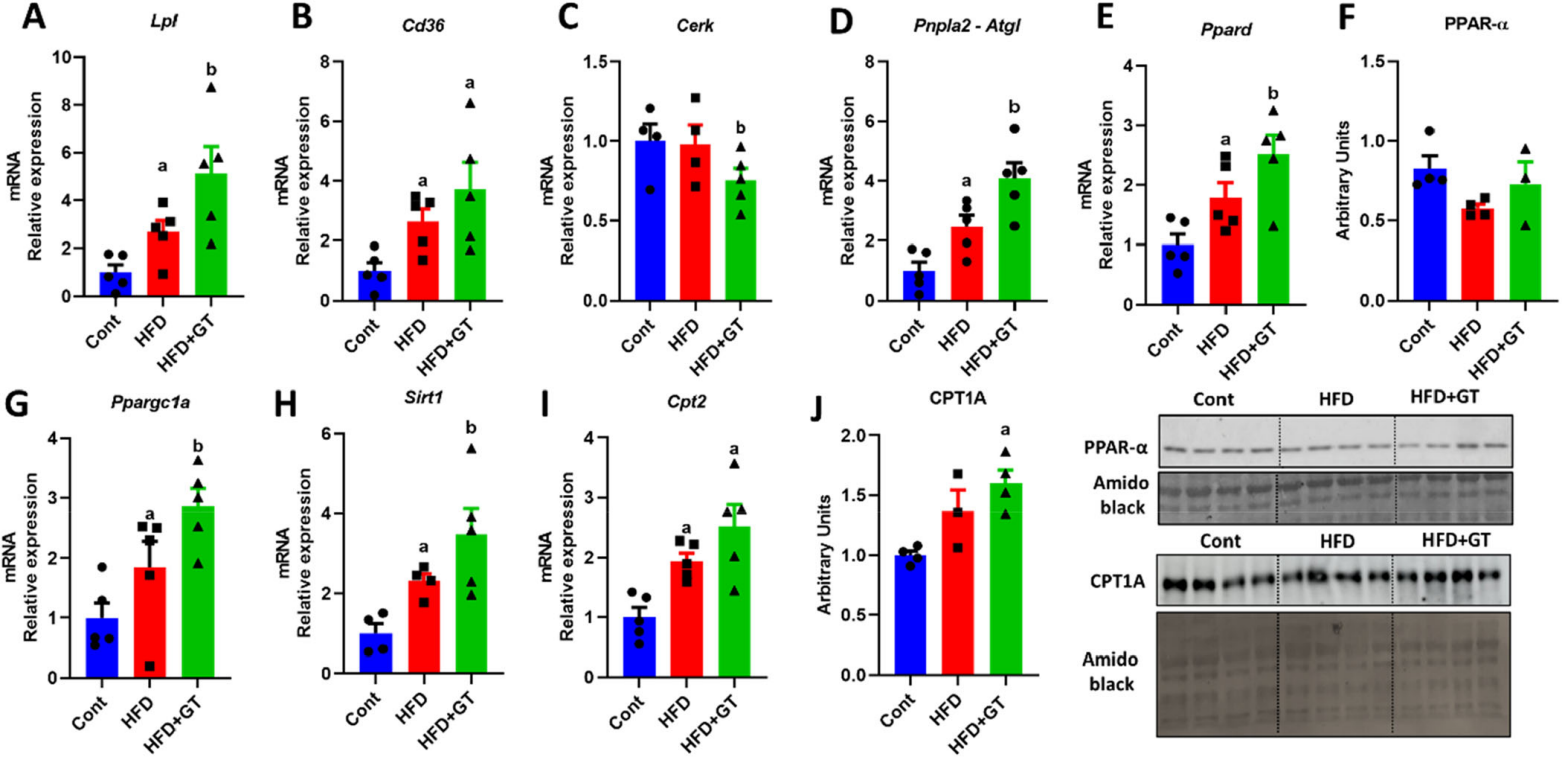
Effect of green tea on lipid metabolism and muscle mitochondrial biogenesis. (A–E) Analysis of gene expression of *Lpl*, *Cd36*, *Cerk*, *Pnpla2*, *Ppard* (variation in relation to control). (F) Evaluation of the amount of PPARα by Western blot (arbitrary units). (G–I) Analysis of gene expression of *Ppargc1a*, *Sirt1*, and *Cpt2* (variation in relation to control). (J) Evaluation of the amount of CPT1A by Western blot (arbitrary units). Results are presented as mean ± SEM of four to five animals per group. Statistical analysis was performed using one‐way ANOVA with Tukey's post hoc test, with a significance level set at *p* < 0.05. Superscript letters indicate: a = statistically significant difference compared with the control group, b = compared with the HFD group.

Under the hypothesis of investigating whether green tea treatment could modulate lipid metabolism in skeletal muscle, we analyzed the expression of some key genes involved in the lipid pathway. An upregulation of lipoprotein lipase *(Lpl)* and *Cd36* genes by both green tea treatment and HFD was observed (Figure 3A,B). Green tea treatment was associated with a reduction in the gene expression of *Cerk* (ceramide kinase – Figure 3C), an enzyme involved in the regulation of lipid metabolism, specifically in the synthesis of ceramides. In addition, a significant increase in the expression of *Pnpla2* (adipose triglyceride lipase) and peroxisome proliferator‐activated receptor delta *(Ppard)* were found in mice treated with green tea (Figure 3D,E). Although we did not observe significant differences in peroxisome proliferator‐activated receptor alpha *(PPARα)* protein content (Figure 3F), there were an increase in the gene expression of Pparg coactivator 1 alpha *(Ppargc1a)* and sirtuin 1 *(Sirt1)* (Figure 3G,H), essential for the biogenesis of mitochondria in skeletal muscle.

In the analysis of lipid oxidation by carnitine palmitoyltransferase 2 *(Cpt2)* gene expression and carnitine palmitoyl transferase 1 (CPT1) protein content, we observed an increase induced by green tea only when compared to the control group (Figure 3I,J, respectively).

**Figure 4:**
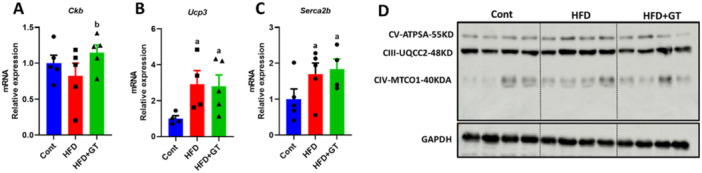
Effect of green tea on skeletal muscle mitochondrial OXPHOS in HFD‐fed mice. (A–C) Analysis of gene expression of *Ckb*, *Ucp3*, and *Serca2b* (variation in relation to control). (D) Evaluation of the amount of OXPHOS by Western blot (arbitrary units). Results are presented as mean ± SEM of four to five animals per group. Statistical analysis was performed using one‐way ANOVA with Tukey's post hoc test, with a significance level set at *p* < 0.05. Superscript letters indicate: a = statistically significant difference compared with the control group, b = compared with the HFD group.

The green tea treatment increased *Ckb* (creatine kinase b) expression in skeletal muscle, suggesting enhanced ATP regeneration during high energy demand, which may support efficient energy expenditure. On the other hand, we did not observe any effect on the modulation of *Ucp3* (uncoupling protein 3), *Serca2b* (ATPase sarcoplasmic/endoplasmic reticulum Ca2+ transporting 2) gene expression, and proteins involved in oxidative phosphorylation (Figure 4A,B). In summary, our results suggest that green tea treatment may influence lipid metabolism and mitochondrial biogenesis in skeletal muscle, enhancing ATP regeneration through increased *Ckb* expression, without significantly affecting other metabolic pathways.

### 3.3 At Thermoneutrality, Green Tea Treatment Improves Insulin Sensitivity and Restores Glucose Tolerance in Obese Mice

**Figure 5:**
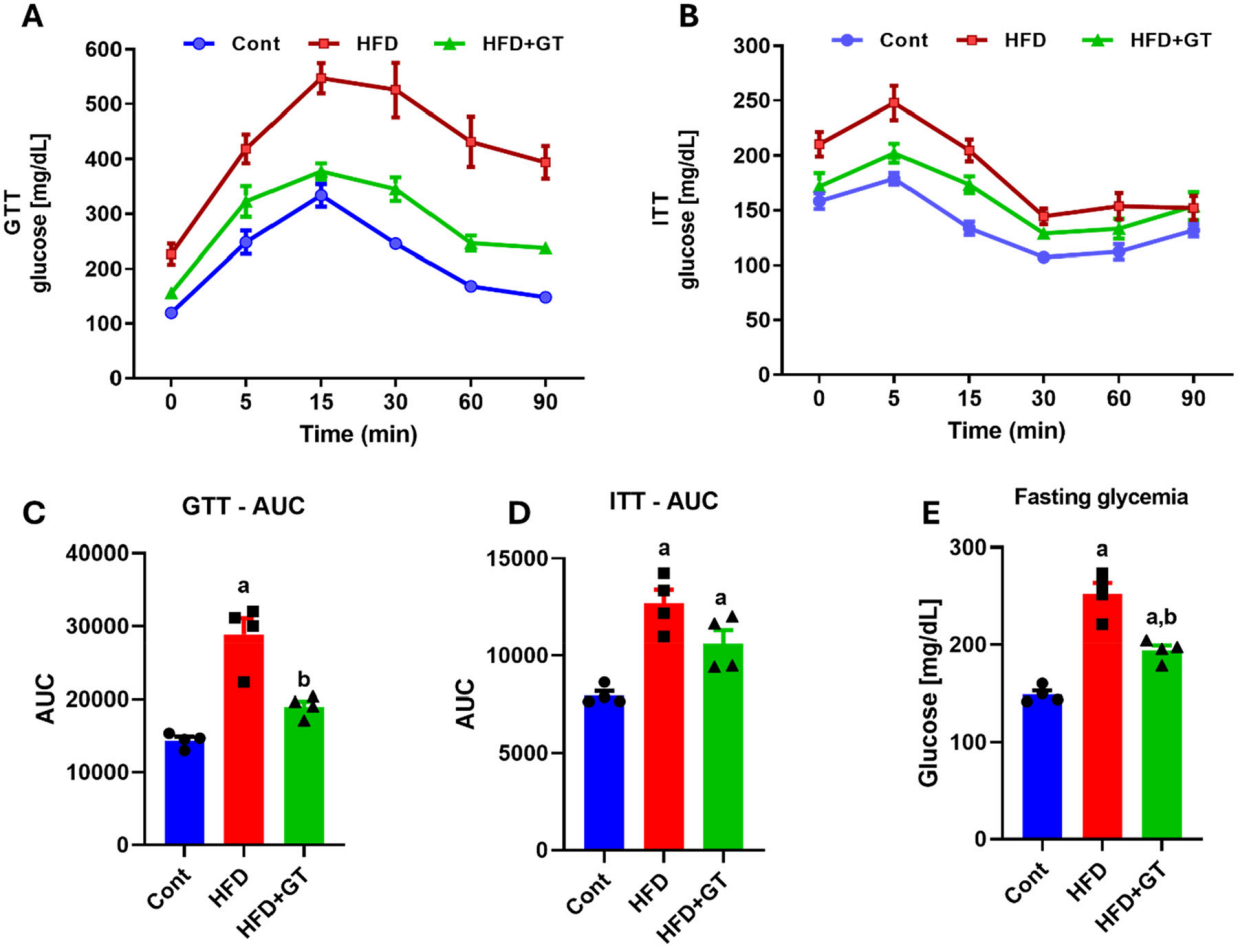
At thermoneutrality, green tea treatment improves insulin sensitivity and restores glucose tolerance in obese mice. (A) Glucose tolerance test (GTT) (glucose mg/dL). (B) Insulin tolerance test (ITT) (glucose mg/dL). (C) Area under the curve (AUC) from GTT. (D) AUC from ITT. (E) Fasting glycemia (glucose mg/dL). Results are presented as mean ± SEM of four to five animals per group. Statistical analysis was performed using one‐way ANOVA with Tukey's post hoc test, with a significance level set at *p* < 0.05. Superscript letters indicate: a = statistically significant difference compared with the control group, b = compared with the HFD group.

The literature has consistently shown the positive impact of green tea extract on glucose regulation. Our findings further validate these benefits, demonstrating that mice treated with green tea exhibit improved insulin sensitivity and glucose tolerance, as indicated by the results of the GTT and ITT (Figure 5A–D). Notably, obese mice showed significantly reduced fasting glucose levels when treated with green tea (Figure 5E), highlighting the protective effects of green tea against systemic insulin resistance in mice on an HFD, even in thermoneutral conditions.

### 3.4 Green Tea Treatment Increases the Expression of Genes Related to Glucose Uptake in Skeletal Muscle

**Figure 6:**
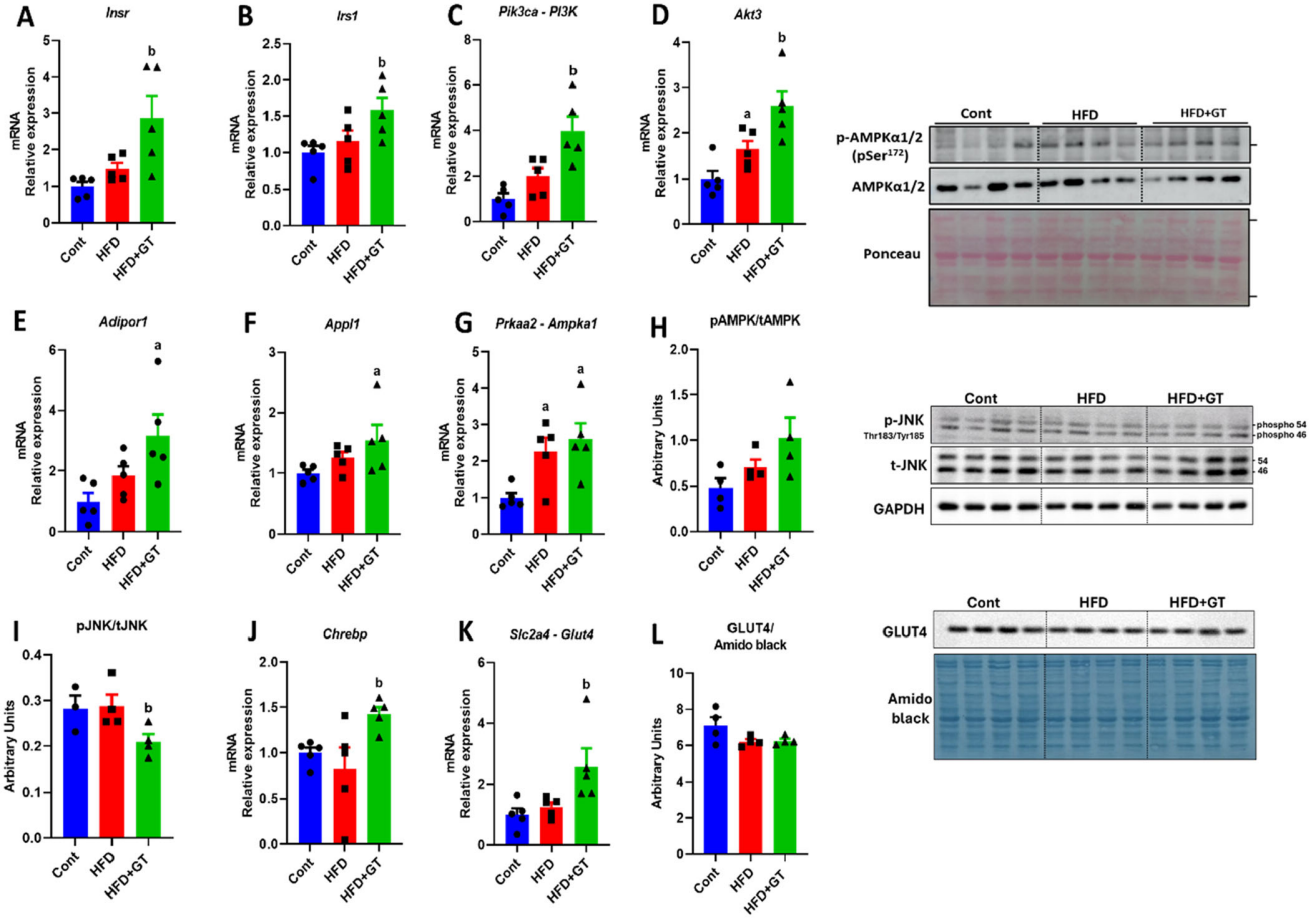
Green tea treatment increases the expression of genes related to glucose uptake in skeletal muscle. (A–D) Analysis of the expression of genes related to the insulin pathway *Insr*, *Irs1*, *Pik3ca* (PI3K), and *Akt3* (variation in relation to the control). (E–G) Analysis of the gene expression of *Adipor1*, *Appl1*, *Prkaa2* (AMPK) (variation in relation to the control group) and (H) protein ratio of pAMPK/AMPK by western blot (arbitrary units). (I) Protein ratio of pJNK/JNK. (J) *Chrebp* gene expression and (K) *Slc2a4* (Glut4) gene expression (variation in relation to the control group). (L) GLUT4 protein content. Results are presented as mean ± SEM of four to five animals per group. Statistical analysis was performed using one‐way ANOVA with Tukey's post hoc test, with a significance level set at *p* < 0.05. Superscript letters indicate: a = statistically significant difference compared with the control group, b = compared with the HFD group.

Our study investigated the potential benefits of green tea treatment for improving insulin sensitivity and glucose handling in obese mice. The results revealed that green tea treatment led to increased gene expression of the insulin receptor (*Insr*) and its substrate (*Insr1*). Additionally, we observed a significant increase in the gene expression of *Pik3ca* (PI3K – phosphoinositide 3‐kinase) and *Akt3* (AKT serine/threonine kinase 3) (Figure 6A–D). However, we did not observe significant changes in the gene expression of *Adipor1* (adiponectin receptor 1), *Appl1* (adaptor protein containing PH domain, PTB domain, and leucine zipper motif 1), *Prkacb* (protein kinase CAMP‐activated catalytic subunit beta), and in the protein content of AMPK (5′ AMP‐activated protein kinase – Figure 6E–H) in the green tea‐treated obese mice compared to obese mice, although all of them were higher compared to the control group. This was associated with a reduction of phosphorylated JNK protein content (Figure 6I). Furthermore, we found an increase in the gene expression of *Chrebp* (Carbohydrate Response Element‐Binding Protein) and *Glut4*, indicating improved glucose uptake (Figure 6J,K). However, mRNA was not reflected in GLUT4 protein content, which was similar among the groups (Figure 6K). Although our findings suggest that green tea influences the insulin signaling pathway, we did not find any changes in the protein levels of GLUT4, even though there was an increase in its mRNA expression. This indicates that while green tea may impact the regulation of the insulin signaling pathway at the transcriptional level, it does not seem to directly affect the protein content of GLUT4.

**Figure 7:**
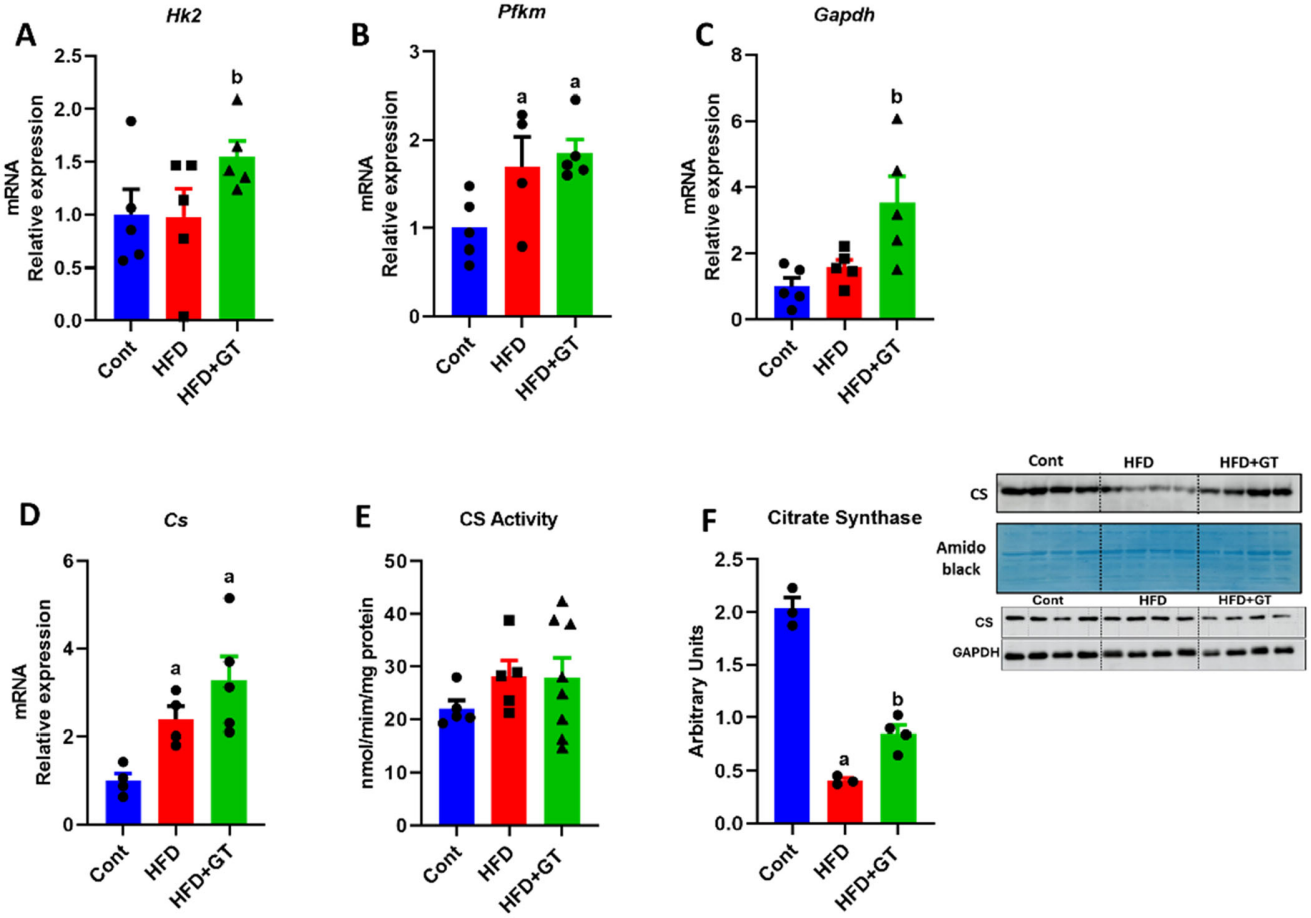
Glucose handling evaluation in skeletal muscle in HFD‐fed mice treated with green tea. (A–D) Analysis of the gene expression of *Hk2*, *Pfkm*, *Gapdh*, and *Cs* (variation in relation to the control). (E) Citrate synthase activity (nmol/min/mg protein). (F) Citrate synthase protein by western blot (arbitrary units). Results are presented as mean ± SEM of four to five animals per group. Statistical analysis was performed using one‐way ANOVA with Tukey's post hoc test, with a significance level set at *p* < 0.05. Superscript letters indicate: a = statistically significant difference compared with the control group, b = compared with the HFD group.

In our study, we observed a significant increase in gene expression of Hexokinase (*Hk*) and glyceraldehyde 3‐phosphate dehydrogenase (*Gapdh*) in mice treated with green tea, indicating a positive impact on glucose metabolism. This suggests that green tea treatment may improve glucose disposal in skeletal muscle. However, the expression of phosphofructokinase muscle (*Pfkm*) did not change in response to the treatment (Figure 7A–C). Furthermore, our analysis of CS gene expression, activity, and protein content revealed some interesting patterns. There was a notable increase in gene expression of CS in all mice fed with an HFD compared to the control group (Figure 7D). Additionally, we observed an increase in CS protein content induced by green tea treatment in HFD‐fed mice. Despite this, we observed a reduction in CS activity in obese mice compared to the control group, and a decrease in CS activity induced by green tea treatment compared to the HFD group (Figure 7E,F). These findings collectively suggest that green tea treatment positively influences gene expression related to glucose disposal in skeletal muscle.

### 3.5 Green Tea Treatment Increases LDH Activity

**Figure 8:**
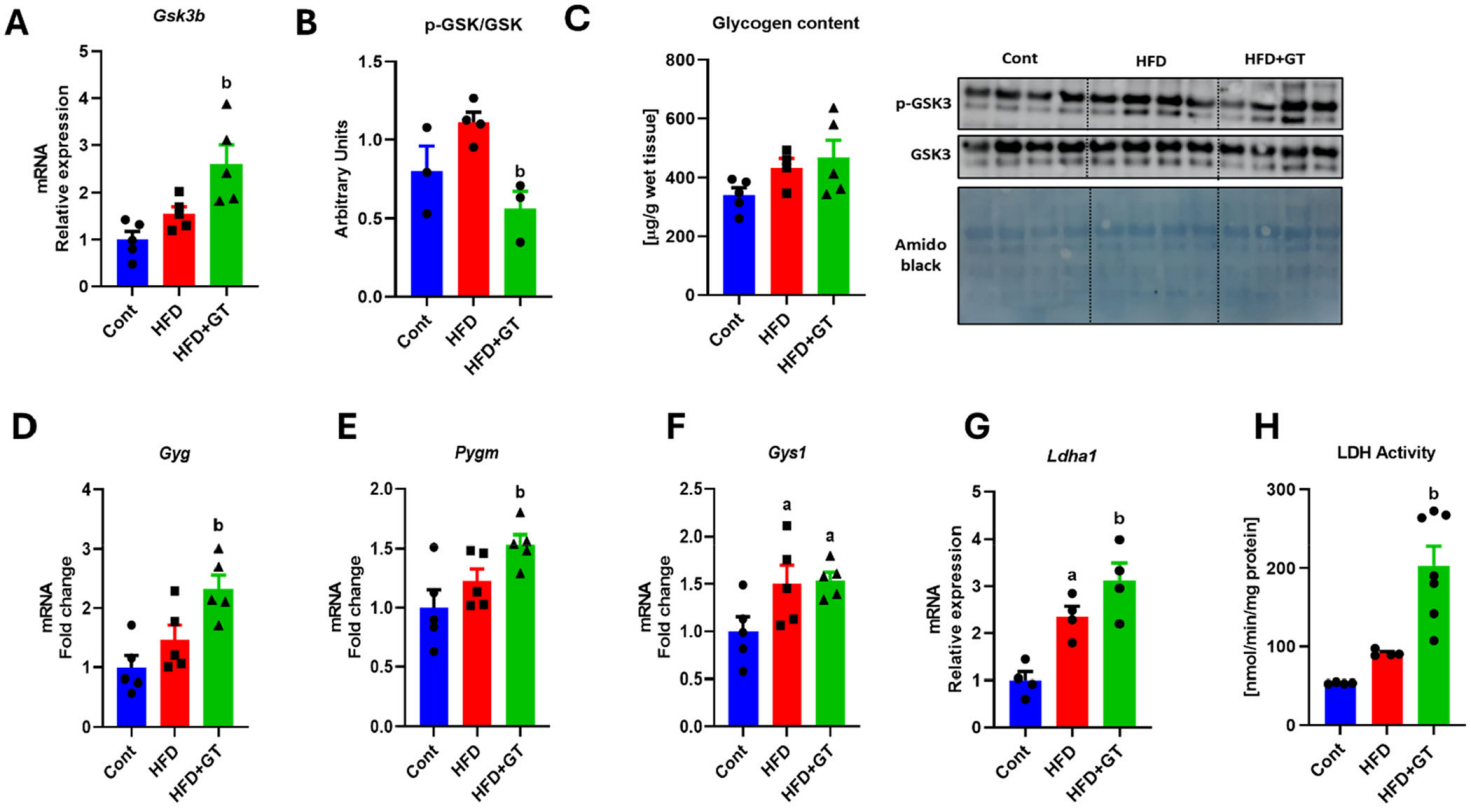
Green tea treatment increases lactate dehydrogenase activity. (A and B) Analysis of the gene expression of *Gsk3b* and protein content of p‐GSK/GSK, respectively (variation with the control). (C) Analysis of glycogen content in gastrocnemius muscle (μg/g of wet tissue). (D–G) Analysis of the gene expression of *Gyg*, *Pygm*, *Gys1*, and *Ldha1* (variation with the control). (H) Lactate dehydrogenase activity (nmol/min/mg protein). Results are presented as mean ± SEM of four to five animals per group. Statistical analysis was performed using one‐way ANOVA with Tukey's post hoc test, with a significance level set at *p* < 0.05. Superscript letters indicate: a = statistically significant difference compared with the control group, b = compared with the HFD group.

In conditions of high energy demand, the Cori cycle in skeletal muscle plays a crucial role in regulating lactate and glucose metabolism, ensuring a constant energy supply to muscle cells. This intricate metabolic process facilitates the conversion of lactate to glucose, thus maintaining energy balance during periods of elevated demand. When obese mice were treated with an HFD and then given green tea, notable changes were observed. The treatment led to increased expression of the *Gsk3b* (glycogen synthase kinase 3) (Figure 8A) gene and a reduction in the pGSK/GSK protein ratio, despite no significant change in intramuscular glycogen levels (Figure 8B–D). Furthermore, there was a noticeable increase in the gene expression of glycogenin (*Gyg*) and glycogen phosphorylase (*Pygm*) in obese mice treated with green tea compared to those on an HFD alone. On the other hand, the expression of *Gys1* was solely induced by the HFD. These data were associated with increased *Ldha1* (lactate dehydrogenase 1a) gene expression and LDH activity (Figure 8G,H). These compelling observations suggest that green tea treatment promotes heightened glycogen synthesis and lactate release in skeletal muscle.

## 4 Discussion

We demonstrate that green tea treatment in HFD‐fed mice improves insulin sensitivity, stimulates lipid metabolism pathways, and enhances glucose utilization, as previously shown in several studies using mouse models of diet‐induced obesity [25, 26, 27, 28]. Previous studies have suggested that green tea may affect body composition and muscle function in obese conditions, but it is not yet clear if these effects are the same in mice kept at thermoneutrality. One important factor to consider when applying these findings to humans is the temperature at which the animals are housed during the study [15, 16]. By maintaining the mice in a thermoneutral zone, where no extra energy is spent on heat production, we ensure that the metabolic changes observed are directly attributable to the interventions under study, such as the effect of green tea. This approach allows for a more reliable evaluation of how green tea affects muscle physiology. Thus, the use of thermoneutral conditions enhances the validity of our findings, allowing us to isolate the effects of the green tea treatment from other environmental factors that might influence metabolic processes. Our study highlights a limitation regarding sexual dimorphism: we conducted our experiments solely on male mice and did not include female mice.

Skeletal muscle is a highly adaptable tissue in mammals, capable of responding to various factors such as heat stress, energy sources, and hormonal signals [29, 30]. It plays a crucial role in metabolic adjustments, particularly in conditions like obesity and insulin resistance, and is vital for maintaining the body's balance, accounting for over 75% of insulin‐mediated glucose disposal [31]. Changes in skeletal muscle development and function are key contributors to the development of obesity, type 2 diabetes, and other metabolic disorders, ultimately leading to cardiometabolic complications.

### 4.1 Effect of Green Tea Treatment on Muscle Function in HFD‐Fed Mice Maintained at Thermoneutrality

In our study, we found no significant changes in the weight, total protein content, and protein density of the gastrocnemius muscle following green tea treatment. Additionally, no notable alterations were observed in the proteins related to muscle function. It is crucial to recognize that the limitations in measuring lean mass using magnetic resonance imaging may warrant additional analysis to substantiate these results. The following study looked at the impact of green tea supplementation on skeletal muscle health, including strength, structure, and the number of muscle stem cells, over a 4–5‐week period [32]. The results showed that green tea supplementation increased antioxidant levels in the blood and reduced muscle damage in mdx mice [32]. Furthermore, an additional study by Onishi et al. [33] showcased that an HFD exacerbated age‐related muscle loss and insulin resistance in obese mice, while green tea intake improved insulin signaling and attenuated muscle weight loss in these senescent, HFD‐fed mice. These findings underscore the necessity of conducting targeted investigations focused on muscle mass in obese mice maintained at a consistent ambient temperature, as thermoneutrality.

### 4.2 Therapeutic Effect of Green Tea on Lipid Metabolism

As previously mentioned, skeletal muscle plays a crucial role in the body's energy metabolism by taking up, storing, and utilizing lipids as an energy source during muscle contraction. Our recent study aimed to investigate the effects of green tea treatment on the expression of various genes associated with lipid metabolism and mitochondrial biogenesis in skeletal muscle. Our compelling findings suggest that green tea treatment may have a significantly positive impact on lipid uptake in skeletal muscle. The increased gene expression of *Lpl* and *Cd36*, genes responsible for transporting and absorbing fatty acids, indicates an enhanced lipid uptake in skeletal muscle. However, while gene expression changes provide metabolic shifts, this study does not directly establish causality between these changes and improvements in insulin sensitivity. This highlights the complexity of interpreting gene expression changes, as alterations at the transcriptional level may reflect a balance of opposing regulatory pathways. These findings, while valuable, are part of a broader network of metabolic regulation, and further investigation, including functional assays and more direct measurements of insulin sensitivity, is necessary to establish a causal relationship and fully elucidate the role of these gene expression changes in modulating insulin sensitivity. Furthermore, the noteworthy decrease in the gene expression of *Cerk*, a gene associated with ceramide metabolism, implies a potential reduction in ceramide production, leading to improved lipid metabolism and reduced inflammation in skeletal muscle.

We observed a significant increase in the gene expression of *Ppargc1a* and *Sirt1*, both of which are genes that are upregulated during mitochondrial biogenesis. Pgc1a participates in the regulation of mitochondrial biogenesis, stimulating the synthesis of new mitochondria and enhancing the oxidative capacity of skeletal muscle [34]. *Sirt1*, on the other hand, regulates energy metabolism by promoting fatty acid oxidation and improving mitochondrial function [35]. The surge in the expression of these genes may indicate an enhancement in the oxidative capacity and energy metabolism of skeletal muscle in response to green tea treatment. However, we did not observe significant modulations in the expression of genes related to lipid oxidation, such as *Cpt2* and PPARα. Additionally, there were no changes in the expression of genes related to the protein complex responsible for oxidative phosphorylation, a critical step in ATP production in mitochondria [36]. Collectively, these results suggest that green tea treatment may positively modulate lipid uptake in skeletal muscle by increasing the expression of genes linked to mitochondrial biogenesis and energy metabolism.

### 4.3 Green Tea Treatment Enhances Insulin Sensitivity and Glucose Disposal in HFD‐Fed Mice Even at Thermoneutrality

The findings of this study indicate that green tea treatment may have a significant impact on insulin sensibility in skeletal muscle. Our observations revealed that green tea positively influences the expression of key genes involved in the insulin signaling pathway, such as the insulin receptor and its substrate (*Irs1*), as well as promoting the gene expression of the glucose transporter (Glut4/*Slc2a4*) and *Hk*, despite not altering GLUT4 protein content. Additionally, the treatment with green tea reduces phosphorylated JNK protein content in HFD‐fed mice. These results align with our previous evidence supporting green tea's potential in enhancing insulin sensitivity and glycaemic control, as documented in earlier studies [13, 17]. The upregulation of genes related to glucose uptake and insulin signaling pathways following green tea treatment highlights its potential as a supplementary approach for managing insulin resistance and type 2 diabetes. An important limitation of our study is that gene expression is not always reflected in protein expression, so these data must be analyzed cautiously. Our previous data demonstrate that green tea protects mice against metabolic‐associated fatty liver disease. We found that green tea enhances insulin sensitivity regardless of housing temperature—whether at standard temperature (22°C) or thermoneutrality (28°C). Importantly, the gene expression patterns related to insulin response in green tea‐supplemented mice closely resembled those in control mice, regardless of temperature conditions [37]. These results support the hypothesis that green tea supplementation maintains body weight and metabolic parameters similar to those of control mice, indicating that insulin signaling genes are kept at baseline levels.

### 4.4 Green Tea Treatment: Impact on LDH Activity and Glycogen Metabolism

The rise in LDH activity suggests an enhancement in glycogen metabolism and lactate release by skeletal muscle as a result of the green tea treatment in HFD‐fed mice. These results are in line with previous studies indicating the stimulating effects of green tea on energy metabolism and energy production by skeletal muscle [38]. Notably, a recent study by Cai et al. (2022) has validated that moderate l‐lactate administration in HFD‐fed mice can effectively suppress pro‐inflammatory macrophage polarization, thus improving insulin resistance in these mice [39]. Immune cell infiltration in tissues, such as skeletal muscle and adipose tissue, plays a crucial role in insulin resistance [40, 41]. We demonstrated that green tea modifies the immune cell profile in adipose tissue in obese mice kept at thermoneutrality, helping to prevent insulin resistance. However, we hypothesize that green tea may also reduce inflammation in skeletal muscle in these conditions. This hypothesis needs to be explored in future studies. These findings underscore the potential of LDH activation to contribute to improved physical performance and exercise capacity in individuals undergoing green tea treatment. Additionally, we have observed a positive modulation in the expression of genes related to glycogen degradation in skeletal muscle, such as *Pygm*. During physical activity, skeletal muscle utilizes both circulating plasma glucose and stored glycogen as fuel sources. Furthermore, it has been demonstrated that endurance exercise promotes the transport of dietary fat towards oxidation rather than storage, in both lean individuals and those who are overweight, due to increased capacity for fatty acid transport in skeletal muscle combined with heightened mitochondrial fatty acid oxidation [42].

## 5 Conclusion

The compelling evidence indicates that administering green tea to mice on an HFD under thermoneutral temperature conditions leads to an enhancement in insulin sensitivity. This remarkable effect is attributed to the upregulation of genes and proteins associated with glucose uptake and disposal in skeletal muscle. These findings suggest that the treatment with green tea improves systemic insulin sensitivity in obese conditions, primarily through its influential impact on skeletal muscle, even in mice maintained at thermoneutrality.

## Author Contributions

C.P.B.S.‐F., V.S., K.L., and R.O. conceived and designed the experiments. C.P.B.S.‐F., M.A.S., A.V., V.S., and K.L. conducted experiments and data analyses. C.P.B.S.‐F. and R.O. conducted manuscript preparation. C.P.B.S.‐F., V.S., and R.O. reviewed the manuscript. All authors approved the final version of this manuscript.

## Conflicts of Interest

The authors declare no conflicts of interest.

## Supporting information

Table 1 ‐ Copia.JPG.

Table 2 ‐ Copia.JPG.

## Data Availability

The data that support the findings of this study are available from the corresponding author upon reasonable request.

## References

[1] A. Sarma , R. Bania , and M. K. Das , “Green Tea: Current Trends and Prospects in Nutraceutical and Pharmaceutical Aspects,” Journal of Herbal Medicine 41 (2023): 100694, 10.1016/J.HERMED.2023.100694.

[2] T. Ohishi , R. Fukutomi , Y. Shoji , S. Goto , and M. Isemura , “The Beneficial Effects of Principal Polyphenols From Green Tea, Coffee, Wine, and Curry on Obesity,” Molecules 26, no. 2 (2021): 453, 10.3390/MOLECULES26020453. 33467101 PMC7830344

[3] P. Li , A. Liu , W. Xiong , et al., “Catechins Enhance Skeletal Muscle Performance,” Critical Reviews in Food Science and Nutrition 60, no. 3 (2020): 515–528, 10.1080/10408398.2018.1549534. 30633538

[4] J. H. Park , J. H. Bae , S. S. Im , and D. K. Song , “Green Tea and Type 2 Diabetes,” Integrative Medicine Research 3, no. 1 (2014): 4–10, 10.1016/J.IMR.2013.12.002. 28664072 PMC5481694

[5] M. Ueda‐Wakagi , H. Nagayasu , Y. Yamashita , and H. Ashida , “Green Tea Ameliorates Hyperglycemia by Promoting the Translocation of Glucose Transporter 4 in the Skeletal Muscle of Diabetic Rodents,” International Journal of Molecular Sciences 20, no. 10 (2019): 2436, 10.3390/IJMS20102436. 31100973 PMC6566303

[6] H. Ma , B. Zhang , Y. Hu , et al., “The Novel Intervention Effect of Cold Green Tea Beverage on High‐Fat Diet Induced Obesity in Mice,” Journal of Functional Foods 75 (2020): 104279, 10.1016/J.JFF.2020.104279.

[7] Y. Wang , H. Xia , J. Yu , et al., “Effects of Green Tea Catechin on the Blood Pressure and Lipids in Overweight and Obese Population—A Meta‐Analysis,” Heliyon 9, no. 11 (2023): e21228, 10.1016/J.HELIYON.2023.E21228. 38034724 PMC10681946

[8] J. Yan , Y. Zhao , S. Suo , Y. Liu , and B. Zhao , “Green Tea Catechins Ameliorate Adipose Insulin Resistance by Improving Oxidative Stress,” Free Radical Biology and Medicine 52, no. 9 (2012): 1648–1657, 10.1016/J.FREERADBIOMED.2012.01.033. 22330066

[9] M. De la Fuente‐Muñoz , M. De la Fuente‐Fernández , M. Román‐Carmena , et al., “Supplementation With a New Standardized Extract of Green and Black Tea Exerts Antiadipogenic Effects and Prevents Insulin Resistance in Mice With Metabolic Syndrome,” International Journal of Molecular Sciences 24, no. 10 (2023): 8521, 10.3390/IJMS24108521/S1. 37239868 PMC10218622

[10] N. Molina , A. P. Bolin , and R. Otton , “Green Tea Polyphenols Change the Profile of Inflammatory Cytokine Release From Lymphocytes of Obese and Lean Rats and Protect Against Oxidative Damage,” International Immunopharmacology 28, no. 2 (2015): 985–996, 10.1016/J.INTIMP.2015.08.011. 26299975

[11] K. F. F. S. Albuquerque , M. P. Marinovic , A. C. Morandi , A. P. Bolin , and R. Otton , “Green Tea Polyphenol Extract In Vivo Attenuates Inflammatory Features of Neutrophils From Obese Rats,” European Journal of Nutrition 55, no. 3 (2016): 1261–1274, 10.1007/S00394-015-0940-Z/METRICS. 26031433

[12] M. P. Marinovic , A. C. Morandi , and R. Otton , “Green Tea Catechins Alone or in Combination Alter Functional Parameters of Human Neutrophils via Suppressing the Activation of TLR‐4/NFκB p65 Signal Pathway,” Toxicology In Vitro 29, no. 7 (2015): 1766–1778, 10.1016/J.TIV.2015.07.014. 26187476

[13] A. P. Bolin , C. P. B. Sousa‐Filho , G. T. N. dos Santos , et al., “Adipogenic Commitment Induced by Green Tea Polyphenols Remodel Adipocytes to a Thermogenic Phenotype,” Journal of Nutritional Biochemistry 83 (2020): 108429, 10.1016/J.JNUTBIO.2020.108429. 32563802

[14] G. L. McKie and D. C. Wright , “The Confounding Effects of Sub‐Thermoneutral Housing Temperatures on Aerobic Exercise‐Induced Adaptations in Mouse Subcutaneous White Adipose Tissue,” Biology Letters 17, no. 6 (2021): 20210171, 10.1098/RSBL.2021.0171. 34186002 PMC8241485

[15] A. W. Fischer , B. Cannon , and J. Nedergaard , “The Answer to the Question “What Is the Best Housing Temperature to Translate Mouse Experiments to Humans?” Is: Thermoneutrality,” Molecular Metabolism 26 (2019): 1–3, 10.1016/J.MOLMET.2019.05.006. 31155502 PMC6667698

[16] J. M. Jacobsen , N. Petersen , L. Torz , et al., “Housing Mice Near vs. Below Thermoneutrality Affects Drug‐Induced Weight Loss but Does Not Improve Prediction of Efficacy in Humans,” Cell Reports 43, no. 8 (2024): 114501, 10.1016/J.CELREP.2024.114501. 39067024 PMC11380917

[17] K. Tognolli , V. Silva , C. P. B. Sousa‐Filho , C. A. L. Cardoso , R. Gorjão , and R. Otton , “Green Tea Beneficial Effects Involve Changes in the Profile of Immune Cells in the Adipose Tissue of Obese Mice,” European Journal of Nutrition 62, no. 1 (2023): 321–336, 10.1007/S00394-022-02963-3. 35994086

[18] C. P. B. Sousa‐Filho , H. O. F. Faria , J. C. Esposito , A. Melo , M. O. Ribeiro , and R. Otton , “Green Tea Improves the Metabolism of Peripheral Tissues in β3‐Adrenergic Receptor‐Knockout Mice,” Pharmacological Research 159 (2020): 104956, 10.1016/J.PHRS.2020.104956. 32480000

[19] L. F. Torres , B. Cogliati , and R. Otton , “Green Tea Prevents NAFLD by Modulation of miR‐34a and miR‐194 Expression in a High‐Fat Diet Mouse Model,” Oxidative Medicine and Cellular Longevity 2019 (2019): 1–18, 10.1155/2019/4168380. PMC6914886 31885789

[20] M. DuBois , K. A. Gilles , J. K. Hamilton , et al., “Colorimetric Method for Determination of Sugars and Related Substances,” Analytical Chemistry 28 (2002): 350–356, 10.1021/AC60111A017.

[21] G. Cooney , R. Curi , A. Mitchelson , P. Newsholme , M. Simpson , and E. A. Newsholme , “Activities of Some Key Enzymes of Carbohydrate, Ketone Body, Adenosine and Glutamine Metabolism in Liver, and Brown and White Adipose Tissues of the Rat,” Biochemical and Biophysical Research Communications 138, no. 2 (1986): 687–692, 10.1016/S0006-291X(86)80551-4. 3741427

[22] B. A. Victor Zammit and E. A. Newsholmet , “The Maimum Activities of Hexo inase, Phosphorylase, Phosphofructokinase, Glycerol Phosphate Dehydrogenases, Lactate Dehydrogenase, Octopine Dehydrogenase, Phosphoenolpyruvate Carboxykinase, Nucleoside Diphosphatekinase, Glutamate‐Oxaloacetate Transaminase and Arginine Kinase in Relation to Carbohydrate Utilization in Muscles From Marine Invertebrates,” Biochemical Journal 160 (1976): 447–462. 13783 10.1042/bj1600447 PMC1164260

[23] R. Otton and R. Curi , “Diabetes Causes Marked Changes in Lymphocyte Metabolism,” Journal of Endocrinology 174, no. 1 (2002): 55–61, 10.1677/JOE.0.1740055. 12098663

[24] M. Bradford , “A Rapid and Sensitive Method for the Quantitation of Microgram Quantities of Protein Utilizing the Principle of Protein‐Dye Binding,” Analytical Biochemistry 72, no. 1/2 (1976): 248–254, 10.1006/ABIO.1976.9999. 942051

[25] A. P. Bolin , C. P. B. Sousa‐Filho , M. P. Marinovic , A. C. Rodrigues , and R. Otton , “Polyphenol‐Rich Green Tea Extract Induces Thermogenesis in Mice by a Mechanism Dependent on Adiponectin Signaling,” Journal of Nutritional Biochemistry 78 (2020): 108322, 10.1016/j.jnutbio.2019.108322. 32120266

[26] M. P. Marinovic , C. P. B. Sousa‐Filho , F. A. H. Batista , et al., “Green Tea Extract Increases Adiponectin and PPAR α Levels to Improve Hepatic Steatosis,” Journal of Nutritional Biochemistry 103 (2022): 108957, 10.1016/J.JNUTBIO.2022.108957. 35134507

[27] C. P. B. Sousa‐Filho , H. O. F. Faria , J. C. Esposito , A. Melo , M. O. Ribeiro , and R. Otton , “Green Tea Improves the Metabolism of Peripheral Tissues in β3‐Adrenergic Receptor‐Knockout Mice,” Pharmacological Research 159 (2020): 104956, 10.1016/J.PHRS.2020.104956. 32480000

[28] R. Otton , A. P. Bolin , L. T. Ferreira , M. P. Marinovic , A. L. S. Rocha , and M. A. Mori , “Polyphenol‐Rich Green Tea Extract Improves Adipose Tissue Metabolism by Down‐Regulating miR‐335 Expression and Mitigating Insulin Resistance and Inflammation,” Journal of Nutritional Biochemistry 57 (2018): 170–179, 10.1016/J.JNUTBIO.2018.03.024. 29734116

[29] A. Pratesi , “Skeletal Muscle: An Endocrine Organ,” Clinical Cases in Mineral and Bone Metabolism 10, no. 1 (2013): 11–14, 10.11138/CCMBM/2013.10.1.011. 23858303 PMC3710002

[30] A. Feraco , S. Gorini , A. Armani , E. Camajani , M. Rizzo , and M. Caprio , “Exploring the Role of Skeletal Muscle in Insulin Resistance: Lessons From Cultured Cells to Animal Models,” International Journal of Molecular Sciences 22, no. 17 (2021): 9327, 10.3390/IJMS22179327. 34502235 PMC8430804

[31] R. A. DeFronzo , “Pathogenesis of Type 2 Diabetes Mellitus,” Medical Clinics of North America 88, no. 4 (2004): 787–835, 10.1016/j.mcna.2004.04.013. 15308380

[32] N. P. Evans , J. A. Call , J. Bassaganya‐Riera , J. L. Robertson , and R. W. Grange , “Green Tea Extract Decreases Muscle Pathology and NF‐κB Immunostaining in Regenerating Muscle Fibers of Mdx Mice,” Clinical Nutrition 29, no. 3 (2010): 391–398, 10.1016/J.CLNU.2009.10.001. 19897286 PMC2882522

[33] S. Onishi , M. Ishino , H. Kitazawa , et al., “Green Tea Extracts Ameliorate High‐Fat Diet–Induced Muscle Atrophy in Senescence‐Accelerated Mouse Prone‐8 Mice,” PLoS One 13, no. 4 (2018): e0195753, 10.1371/JOURNAL.PONE.0195753. 29630667 PMC5891070

[34] C. Kang and L. Li Ji , “Role of PGC‐1α Signaling in Skeletal Muscle Health and Disease,” Annals of the New York Academy of Sciences 1271, no. 1 (2012): 110–117, 10.1111/J.1749-6632.2012.06738.X. 23050972 PMC3499658

[35] P. S. Pardo and A. M. Boriek , “The Physiological Roles of Sirt1 in Skeletal Muscle,” Aging 3, no. 4 (2011): 430–437, 10.18632/AGING.100312. 21483036 PMC3117458

[36] B. Korzeniewski and H. B. Rossiter , “Each‐Step Activation of Oxidative Phosphorylation Is Necessary to Explain Muscle Metabolic Kinetic Responses to Exercise and Recovery in Humans,” Journal of Physiology 593, no. 24 (2015): 5255–5268, 10.1113/JP271299. 26503399 PMC4704516

[37] V. Silva , H. O. F. Faria , C. P. B. Sousa‐Filho , J. F. R. de Alvarenga , J. Fiamoncini , and R. Otton , “Thermoneutrality or Standard Temperature: Is There an Ideal Housing Temperature to Study the Antisteatotic Effects of Green Tea in Obese Mice?,” Journal of Nutritional Biochemistry 120 (2023): 109411, 10.1016/J.JNUTBIO.2023.109411. 37423321

[38] M. Rondanelli , M. Nichetti , G. Peroni , et al., “Where to Find Leucine in Food and How to Feed Elderly With Sarcopenia in Order to Counteract Loss of Muscle Mass: Practical Advice,” Frontiers in Nutrition 7 (2021): 622391, 10.3389/FNUT.2020.622391. 33585538 PMC7874106

[39] H. Cai , X. Wang , Z. Zhang , et al., “Moderate L‐Lactate Administration Suppresses Adipose Tissue Macrophage M1 Polarization to Alleviate Obesity‐Associated Insulin Resistance,” Journal of Biological Chemistry 298, no. 4 (2022): 101768, 10.1016/J.JBC.2022.101768. 35218776 PMC8941214

[40] T. J. Guzik , D. S. Skiba , R. M. Touyz , and D. G. Harrison , “The Role of Infiltrating Immune Cells in Dysfunctional Adipose Tissue,” Cardiovascular Research 113, no. 9 (2017): 1009–1023, 10.1093/CVR/CVX108. 28838042 PMC5852626

[41] M. K. Lee , H. Ryu , J. Y. Van , et al., “The Role of Macrophage Populations in Skeletal Muscle Insulin Sensitivity: Current Understanding and Implications,” International Journal of Molecular Sciences 24, no. 14 (2023): 11467, 10.3390/IJMS241411467. 37511225 PMC10380189

[42] A. M. Mengeste , A. C. Rustan , and J. Lund , “Skeletal Muscle Energy Metabolism in Obesity,” Obesity 29, no. 10 (2021): 1582–1595, 10.1002/OBY.23227. 34464025

