# Supplementary figures and images for: Does Green Tea Ameliorate Obesity in Mice Kept at Thermoneutrality by Modulating Skeletal Muscle Metabolism?
Source: Cell Biochem Funct. 2025 Jun 16;43(6):e70094. doi: 10.1002/cbf.70094 (PMC12169088; doi:10.1002/cbf.70094)

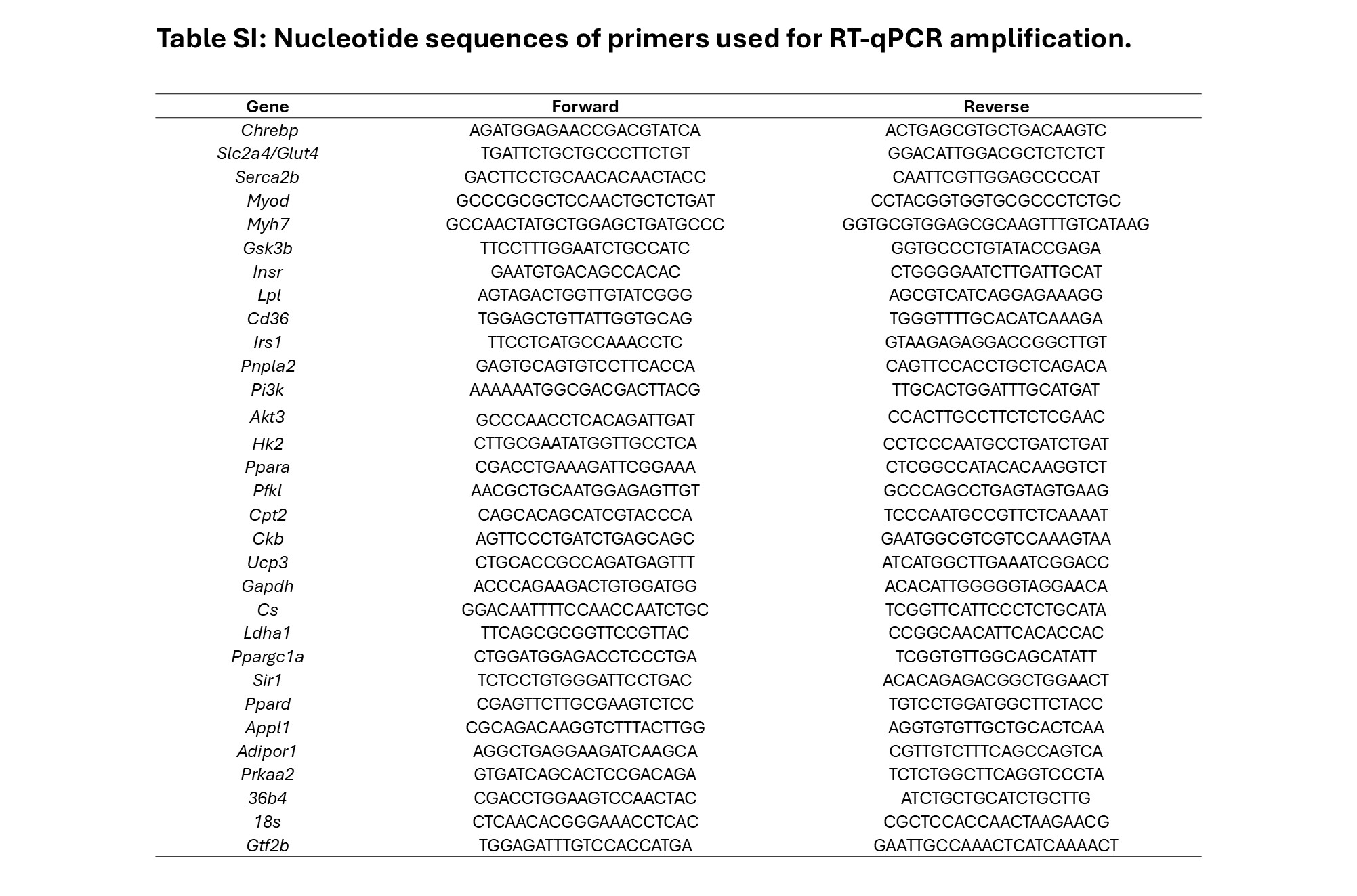

Supplement: Supplementary file 1 — Table 1 ‐ Copia.JPG. [file CBF-43-e70094-s002.JPG]

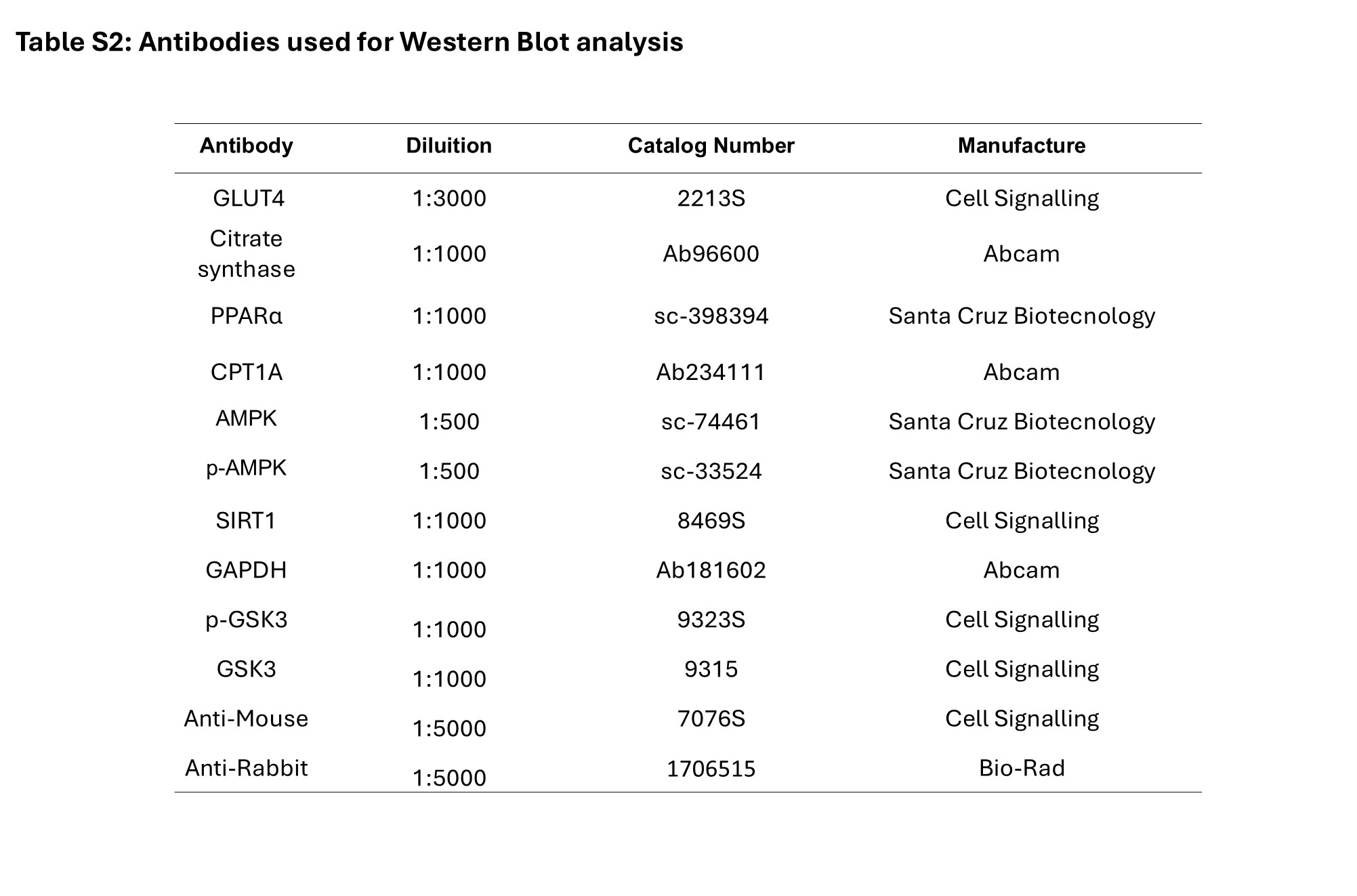

Supplement: Supplementary file 2 — Table 2 ‐ Copia.JPG. [file CBF-43-e70094-s001.JPG]
